# Imaging assessment of toxicity related to immune checkpoint inhibitors

**DOI:** 10.3389/fimmu.2023.1133207

**Published:** 2023-02-23

**Authors:** Antonia M. Berz, Sarah Boughdad, Naïk Vietti-Violi, Antonia Digklia, Clarisse Dromain, Vincent Dunet, Rafael Duran

**Affiliations:** ^1^ Department of Diagnostic and Interventional Radiology, Lausanne University Hospital and University of Lausanne, Lausanne, Switzerland; ^2^ Department of Radiology, Charité – Universitätsmedizin Berlin, Corporate Member of Freie Universität Berlin and Humboldt- Universität zu Berlin, Berlin, Germany; ^3^ Department of Nuclear Medicine and Molecular Imaging, Lausanne University Hospital and University of Lausanne, Lausanne, Switzerland; ^4^ Department of Oncology, Lausanne University Hospital and University of Lausanne, Lausanne, Switzerland

**Keywords:** immunotherapy, immune checkpoint inhibitor, checkpoint inhibition, toxicity, immune-related adverse event, imaging assessment

## Abstract

In recent years, a wide range of cancer immunotherapies have been developed and have become increasingly important in cancer treatment across multiple oncologic diseases. In particular, immune checkpoint inhibitors (ICIs) offer promising options to improve patient outcomes. However, a major limitation of these treatments consists in the development of immune-related adverse events (irAEs) occurring in potentially any organ system and affecting up to 76% of the patients. The most frequent toxicities involve the skin, gastrointestinal tract, and endocrine system. Although mostly manageable, potentially life-threatening events, particularly due to neuro-, cardiac, and pulmonary toxicity, occur in up to 30% and 55% of the patients treated with ICI-monotherapy or -combination therapy, respectively. Imaging, in particular computed tomography (CT), magnetic resonance imaging (MRI), and 2-deoxy-2-[^18^F]fluoro-D-glucose positron emission tomography/computed tomography (^18^F-FDG-PET/CT), plays an important role in the detection and characterization of these irAEs. In some patients, irAEs can even be detected on imaging before the onset of clinical symptoms. In this context, it is particularly important to distinguish irAEs from true disease progression and specific immunotherapy related response patterns, such as pseudoprogression. In addition, there are irAEs which might be easily confused with other pathologies such as infection or metastasis. However, many imaging findings, such as in immune-related pneumonitis, are nonspecific. Thus, accurate diagnosis may be delayed underling the importance for adequate imaging features characterization in the appropriate clinical setting in order to provide timely and efficient patient management. ^18^F-FDG-PET/CT and radiomics have demonstrated to reliably detect these toxicities and potentially have predictive value for identifying patients at risk of developing irAEs. The purpose of this article is to provide a review of the main immunotherapy-related toxicities and discuss their characteristics on imaging.

## Introduction

Immunotherapies, in particular immune checkpoint inhibitors (ICIs), have led to a paradigm shift in cancer treatment in only a few decades and provide promising therapy options across many oncologic diseases (1). The market release of monoclonal antibodies (mABs) targeting the T-lymphocyte-associated protein-4 (anti-CTLA-4) as the first US Food and Drug Administration approved ICI for advanced-stage melanoma in 2011 was followed by the approval of mAbs targeting other ICIs such as programmed cell death protein-1 (anti-PD-1) and PD-1 ligand (anti-PD-L1) (2, 3). Importantly, the sites of action of anti-CTLA-4 and anti-PD-1/PD-L1 antibodies are different. Thus, anti-CTLA-4 acts at the lymph node level at the time of priming, while anti-PD-1/PD-L1 becomes active later in the activation cascade and directly at the tumor site. Their complementary mechanisms of action allow the combined use of these two types of treatment for certain indications (4). Being extensively studied, these novel therapies have demonstrated unprecedented prolongation of patient survival compared with non-ICI treatment (5). This is the case for cancers such as metastatic melanoma, non-small cell lung cancer, renal cell carcinoma, bladder cancer, and refractory Hodgkin’s lymphoma for which only limited treatments options were available before the advent of immune checkpoint blockade (6). Based upon the success gathered by ICIs, many novel molecules are currently being investigated.

However, the unique mechanism of action of ICIs, eliciting a T-cell mediated immune response, has led to two major problems. First, ICI therapy causes specific tumor response patterns, including imaging progression prior to response (pseudoprogression), the paradoxical acceleration of tumor growth kinetics after initiation of immunotherapy (hyperprogression), and the coexistence of responding and non-responding lesions within the same patient (dissociated responses), which are less commonly observed following cytotoxic chemotherapy and targeted therapies (7, 8). These response characteristics lead to a complete revision of the traditionally used Response Evaluation Criteria in Solid Tumors version 1.1 (RECIST 1.1) to accurately assess the tumor response after immunotherapy; the immune-related response criteria (irRC) and subsequently the immune-related RECIST criteria (irRECIST) were introduced (9–11). Second, ICIs can lead to immune-related adverse events (irAEs), which may occur in the majority of patients (up to 76%) with off-target effects potentially affecting any organ system or tissue due to an over activated immune system (12). Several irAE mechanisms have been described, including increasing T-cell activity against antigens present in healthy tissue, upregulation of pre-existing autoantibodies and inflammatory cytokines, as well as enhanced complement-mediated inflammation by direct binding of anti-CTLA-4 antibody’s to CTLA-4 expressed in normal tissue (13). Many of these events are mild and manageable. However, life threatening events, requiring ICI therapy discontinuation, occur in 3%-30% of patients treated with ICI-monotherapy and in up to 55% of patients receiving ICI-combination therapy (12, 14, 15). In clinical practice, it remains a major challenge to detect and adequately address these toxicities. Many hospitals have implemented clinical units specialized in irAEs to ensure optimal patient management. Imaging has also proven to be valuable in this setting as 70% of irAEs can be diagnosed already on ultrasound (US), 79% on computed tomography (CT), 83% on magnetic resonance imaging (MRI) and up to 74% on 2-deoxy-2-[18F]fluoro-D-glucose positron emission tomography/CT (^18^F-FDG-PET/CT) (16). This is all the more interesting as for some patients, irAEs can be detected on imaging even prior to the onset of clinical symptoms (8, 17, 18). This underscores the importance for radiologists and nuclear medicine physicians to become familiar with irAEs and recognize their imaging features, especially as there is some overlap between these toxicities and immunotherapy-related response patterns.

The purpose of this article was to provide a detailed review of main immunotherapy-related toxicities and to discuss their characteristics on imaging.

## Clinical relevance of immune-related adverse events

Immunotherapies, although generally considered to be safer than standard chemotherapies, have a different spectrum of toxicities, most of which are due to excessive immune reactions that can potentially affect any organ system and tissue (13, 19–21). In general, the onset of irAEs is less predictable than for cytotoxic chemotherapy-related side effects which usually appear shortly after treatment initiation. By contrast, the median time from ICI therapy initiation and appearance of irAEs ranges from 2 to 16 weeks but can occur any time during or after the treatment (22, 23). Nonetheless, the risk of irAEs is 3 times higher in the first 4 weeks of treatment, consisting mainly of dermatologic disorders (24, 25). However, delayed irAEs manifesting ≥90 days after discontinuation of immunotherapy may occur in 5.7% of the patients (26, 27).

In general, most frequent irAEs include dermatologic, gastrointestinal, and endocrine toxicities (15, 27, 28). Conversely, neurological, cardiological and pulmonary toxicities have been described as most lethal (15, 29). The occurrence of a certain type of irAEs is highly dependent on a particular drug. A recent meta-analysis, which included 36 head-to-head phase II and III randomized trials, showed that the most common drug-dependent toxicities are hypothyroidism, nausea, and vomiting for atezolizumab (anti-PD-L1 mAb), endocrine toxicities for nivolumab, arthralgia, pneumonitis and hepatic toxicities for pembrolizumab (anti-PD1 mAb), and dermatologic, gastrointestinal and renal toxicities for ipilimumab (anti-CTLA-4 mAb) (12). In addition, the severity of these toxicities also highly depends on the drug target, with atezolizumab (probability 76%, pooled incidence of grade 1-5 adverse events 66.4%) having the best safety profile, followed by nivolumab (56%, 71.8%), pembrolizumab (55%, 75.1%), and ipilimumab (55%, 86.8%) (12). Lethal irAEs occur in 0.37% of anti-PD1, in 0.38% of anti-PD-L1, in 1.08% of anti-CTLA-4, and in 1.23% of patients receiving anti-CTL-4/anti-PD1/PD-L1 combination immunotherapy (15). Risk factors for the development of irAEs are genetics, environmental factors, previous toxicities with immunotherapies, the patient’s own microbiome, and recent severe or chronic viral infections (28). Besides, a systematic review showed that antitumor immune responses and possible toxicity can vary among patients treated with the same ICI depending on the oncologic diseases. Melanoma patients have a significantly higher prevalence of gastrointestinal and skin irAEs, whereas they are less likely to have pneumonitis compared with patients with non-small cell lung cancer. Arthritis and myalgias occur more frequently in melanoma patients than in renal cell cancer, where pneumonitis and dyspnea are more common (30).

Moreover, the combination of different immunotherapies increases the risk, frequency, and severity of side effects significantly (21). These severe irAEs often lead to ICI treatment discontinuation and initiation of immunosuppressive therapy, e.g. with corticosteroids.

In order to compare treatment-related complications in a reproducible manner, the U.S. National Cancer Institute has classified the severity of adverse events in the Common Terminology Criteria for Adverse Events (CTCAE) version 6.0 (**Table 1**) (31). CTCAE facilitates a consistent and reproducible comparison of toxicities across clinical trials and can also be applied in the assessment of irAEs in patients treated with immunotherapy (31). Nevertheless, several studies have reported that the presence and severity of irAEs in patients treated with ICI is associated with treatment response suggesting a good prognostic value (32, 33).

**Table 1:** Common Terminology Criteria for Adverse Events (CTCAE) version 6.0 to classify immune irAEs (31).

Grade	Severity and clinical description
1	Mild: asymptomatic or mild symptoms; clinical or diagnostic observations only; intervention not indicated.
2	Moderate: minimal, local or noninvasive intervention indicated; limiting age-appropriate instrumental ADL.
3	Severe or medically significant but not immediately life-threatening: hospitalization or prolongation of hospitalization indicated; disabling; limiting self-care ADL.
4	Life-threatening consequences: urgent intervention indicated.
5	Death related to the adverse event.

ADL, activity of daily living.

**Table 2** summarizes recommended imaging to be prescribed in the presence of suspected irAEs. In this context, it should be noted that many irAEs can be diagnosed clinically (and/or based on blood testing) without necessarily the need to perform imaging. Moreover, the choice of an imaging modality may vary based on all clinical parameters and patient’s condition, and based on available imaging equipment. **Table 3** summarizes the visibility of irAEs on imaging.

**Table 2:** Recommended imaging to be prescribed in the presence of suspected immune-related adverse events (irAEs).

irAEs	US	CT	MRI	^18^F-FDG PET/CT
Enteritis	–	√	(√)	–
Colitis	–	√	(√)	–
Hepatitis	√	(√)	(√)	–
Cholecystitis and cholangitis	√	(√)	√	–
Pancreatitis	√	√	(√)	–
Acute kidney injury	√	–	(√)	–
Pneumonitis	–	√	–	–
Sarcoid-like reactions	–	√	–	(√)
Myocarditis	√	–	√	(√ *)
Pericarditis	√	–	√	–
Myositis	–	–	√	(√ *)
Encephalitis	–	–	√	(√)
Aseptic meningitis	–	–	√	–
Central nervous system vasculitis	–	(√)	√	(√)
Hypophysitis	–	–	√	√
Thyroid dysfunction	√	–	–	–
Primary adrenal insufficiency or adrenalitis	–	(√)	(√)	–


US, ultrasound; CT, computed tomography; MRI, magnet resonance imaging; ^18^F-FDG PET/CT, 2-deo-y-2-[18F]fluoro-D-glucose positron emission tomography.

√, 1st choice modality; (√), optional imaging; -, usually not prescribed or not applicable; * ^68^Ga-DOTATOC PET/CT as additional option.

**Table 3:** Visibility of immune-related adverse events (irAEs) on imaging.

		Visibility of irAEs on imaging			
irAEs	Best imaging for visualization	US	CT	MRI	^18^F-FDG PET/CT
Enteritis	CT	–	√	(√)	(√)
Colitis	CT	–	√	(√)	(√) *
Hepatitis	US	(√)	(√)	(√)	(√) *
Cholecystitis and cholangitis	US	√	(√)	√	(√)
Pancreatitis	CT	(√)	√	√	√
Acute kidney injury	US	(√)	(√)	(√)	(√) *
Pneumonitis	CT	–	√	–	√
Sarcoid-like reactions	PET/CT	–	√	(√)	√
Myocarditis	MRI - PET/CT	(√)	(√)	√	√ **
Pericarditis	MRI	(√)	(√)	√	√
Myositis	MRI	(√)	(√)	√	√ **
Encephalitis	MRI	–	–	√	√ *
Aseptic meningitis	MRI	–	–	√	–
Central nervous system vasculitis	MRI	–	(√)	√	(√)
Hypophysitis	MRI	–	(√)	√	√
Thyroid dysfunction	US	√	√	√	√
Primary adrenal insufficiency or adrenalitis	MRI	–	√	√	√


US, ultrasound; CT, computed tomography; MRI, magnet resonance imaging; ^18^F-FDG PET/CT, 2-deo-y-2-[18F]fluoro-D-glucose positron emission tomography.

√, good visibility; (√), moderate visibility; -, poor visibility or not applicable; * Decreased visibility due to physiological ^18^F-FDG uptake; ** ^68^Ga-DOTATOC PET/CT as an additional option.

## Imaging of immune-related adverse events

### Abdominal toxicities

**Figure 1:**
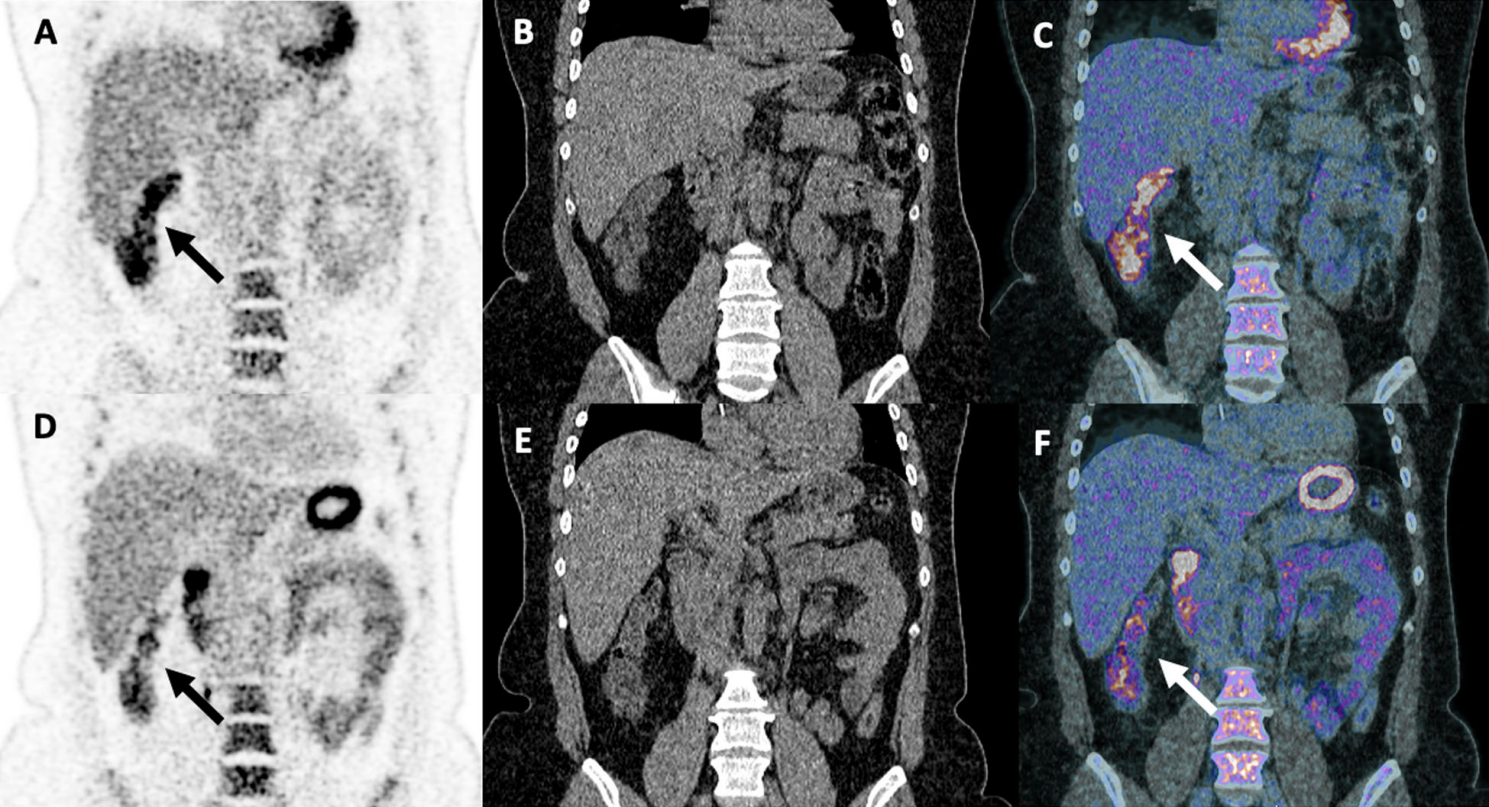
Partial pathological uptake of the right colon suspecting early signs of colitis in a 48-year-old woman with stage IV melanoma treated with two cycles of ipilimumab (anti-CTLA-4) and nivolumab (anti-PD-1). Treatment was discontinued due to grade II colitis diagnosed 3 weeks after the first ^18^F-FDG PET/CT scan [arrows; coronal PET **(A)**, CT **(B)** and merged PET/CT **(C)** images], which required high-dose steroid treatment for 3 months. A decreased right colon uptake was observed on follow-up ^18^F-FDG PET/CT examination performed 4 weeks after the introduction of steroid treatment [arrows; coronal PET **(D)**, CT **(E)** and fused PET/CT images **(F)**].

Diarrhea is one of the most common irAEs, affecting approximately 44% (vs. 10% for grade 3-4) of patients treated with a combination of CTLA-4 and PD-(L)1 inhibitors, 36% (vs. 8% for grade 3-4) of patients treated with anti-CTLA-4, and 11% (vs. 1% for grade 3-4) of patients treated with anti-PD-(L)1 (34). This symptom is often associated with colitis, another common irAE, as it is reported in 16% (vs. 11% for grade 3-4) (combination of CTLA-4 and PD-(L)1 inhibitors), 8% (vs. 5% for grade 3-4) (anti-CTLA-4), and 1% (vs. 1% for grade 3-4) (anti-PD-(L)1) of ICI-treated patients (34). With a median time to full onset of 5 to 10 weeks, colitis can lead to various complications, including intestinal perforation, ischemia, necrosis, hemorrhage, and toxic megacolon (35, 36). Therefore, the typical diagnostic workup in these cases includes contrast-enhanced CT, in which ICI-induced colitis appears as diffuse inflammation with bowel wall thickening (>4mm), mucosal hyperenhancement, mesenteric hyperemia, mesenteric vessel congestion, and air-fluid levels (8, 37). In addition, cases of segmental colitis in association with diverticulosis and isolated rectosigmoid colitis without diverticulosis have been described (38, 39). A recent study including patients with various types of cancer showed CT findings suggestive of colitis in 20 of 34 patients with symptoms of colitis and in 5 of 19 patients even without clinical presentation of colitis (40). ^18^F-FDG PET/CT may reveal increased partial or diffuse tracer uptake in colitis or throughout the entire bowel in patients with extensive inflammation (41, 42) (**Figure 1**). Moreover, it has been reported that it might be more sensitive than CT for the early detection of colitis in patients undergoing ICI treatment (43). However, its lack of specificity for instance due to physiological muscular activity or in patients treated with metformin hinders its value in routine practice (41, 43).

In the context of ICI therapy, hepatitis, characterized by elevation of serum alanine transaminase and/or aspartate transaminase, is often initially clinically asymptomatic but can potentially lead to transient life-threatening liver dysfunction (35). It occurs in 19% (vs. 9% for grade 3-4) of patients treated with ICI-combination therapy, in 5% (vs. 2% for grade 3-4) of patients receiving anti-CTLA-4 and in 19% (vs. 9% for grade 3-4) of patients receiving anti-PD-(L)1 treatment (34). Although hepatic toxicity can occur with a delay of months to years, it typically manifests between 1 and 15 weeks after treatment (36). Recently, in a large population of melanoma patients treated with ipilimumab and/or nivolumab, especially in ICI-combination treatment caused higher rates of grade 3-4 liver toxicity with aminotransferase levels of 6.1% (for aspartate aminotransferase) and 8.3% (for alanine aminotransferase), and have been shown to lead frequently to ICI treatment discontinuation (44). Evidence for hepatitis can be found on US as a diffusely hypoechogenic liver parenchyma with periportal thickening and hyperechogenic dots, known as “starry sky pattern” sign (**Supplementary Figure 1**), as well as gallbladder wall thickening (45). CT and MRI findings are often nonspecific and comparable to those of other causes of acute liver dysfunction, ranging from the absence of detectable abnormalities to hepatomegaly, heterogeneous parenchymal enhancement with areas of low attenuation, periportal/gallbladder edema (diffuse parenchymal hypoattenuation on CT or T2-weighted hyperintensity on MRI), and perihepatic ascites (36, 37, 42, 45). Interestingly, while other causes of diffuse liver disease might not be visualized on PET/CT, increased liver ^18^F-FDG avidity in the setting of ICI-induced hepatitis is often reported (46). However, its visualization using ^18^F-FDG PET/CT might be limited by physiological uptake or a reversal in liver-to-spleen ratio due to higher spleen uptake resulting from ICI-induced T-cell activation especially at an early stage (47). Some authors have suggested the use of a liver-to-blood pool standard uptake value (SUV) mean ratio to detect pathologic hepatic uptake and thus possible hepatitis compared to SUVmean alone because various parameters can influence SUV measurements (48, 49).

**Figure 2:**
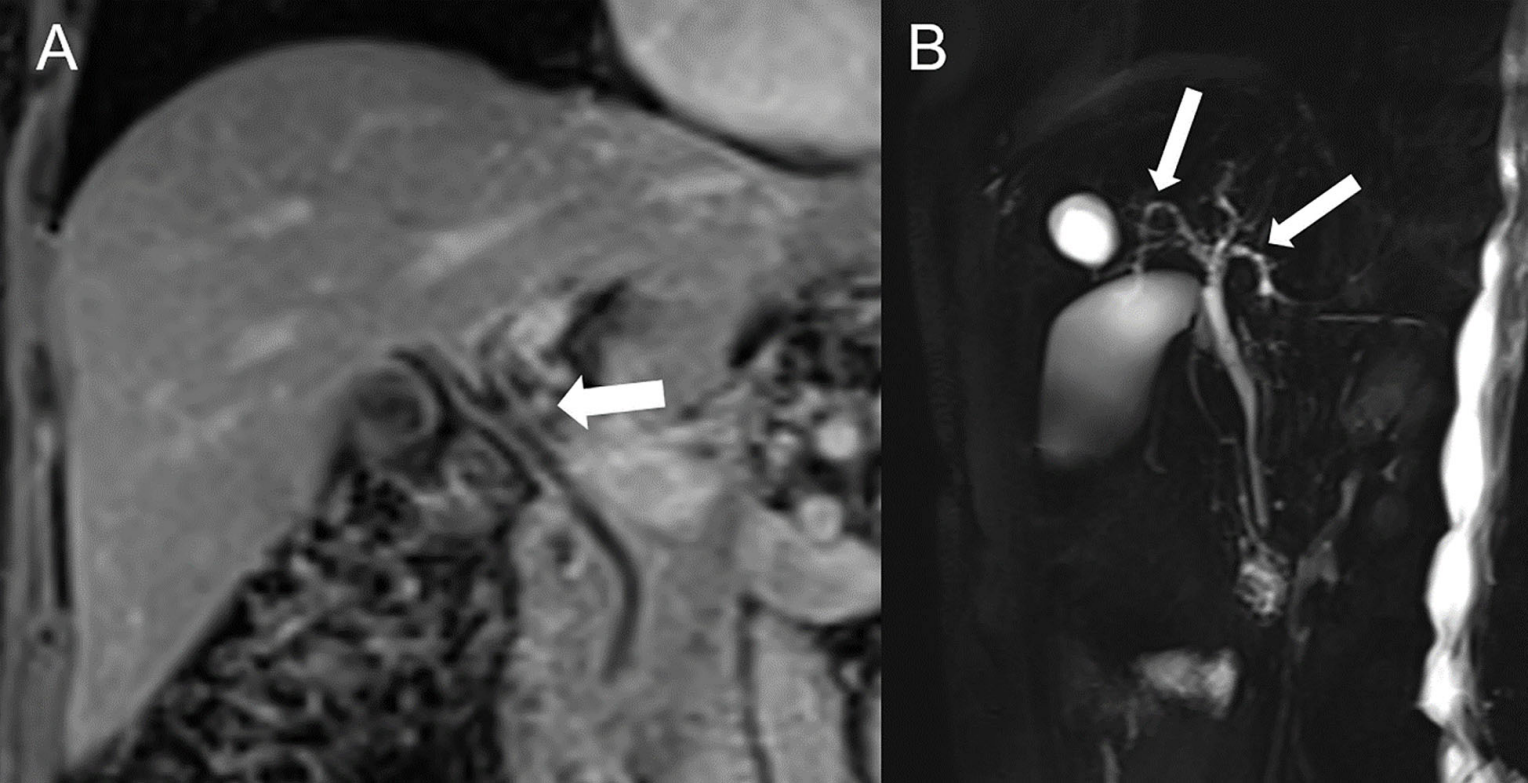
Immune-related hepatitis and cholangitis in a 73-year-old female with metastatic melanoma of the tibia on nivolumab (anti-PD-1) and ipilimumab (anti-CTLA-4) who developed grade 3 hepatitis. **(A)** Contrast-enhanced MRI in coronal view show hyperhemic and slightly thickened bile duct walls (arrow) and **(B)** MR cholangiopancreatography demonstrate bile ducts irregularities (arrows) compatible with immune-mediated cholangitis.

Cholecystitis and cholangitis are forms of hepatobiliary toxicity that are rarely associated with ICI therapy, and because of the small reported number of cases, it is difficult to estimate the actual incidence and causal relationship, if any, with immunotherapy (35, 50, 51). Despite its low incidence characteristic imaging features on US and CT for cholecystitis such as gallbladder distension and wall thickening, as well as inflammation of the pericholecystic tissue with increased ^18^F-FDG uptake on PET/CT, have been suggested (52). On MRI and MR cholangiopancreatography (MRCP), as the imaging modalities of choice in clinical practice, cholangitis is characterized by localized dilatation and diffuse non-obstructive hypertrophy and hyperenhancement of the extrahepatic bile duct wall with portions of biliary dilatation and narrowing (**Figure 2**) (51, 52).

The diagnosis of pancreatitis requires the presence of at least two of the following three features: abdominal pain suggestive of pancreatitis, elevated amylase or lipase to more than three times of the upper normal limit, and characteristic imaging features (53) because ICI-related acute pancreatitis is relatively rare and patients with elevated amylase and lipase are often initially asymptomatic, only few cases have been reported (54). Radiologic findings on CT and MRI are similar to pancreatitis from other origin and include in the acute phase focal or diffuse pancreatic enlargement with decreased enhancement and peripancreatic fat stranding associated with edema and fluid collections without a focal lesion suspicious for metastasis (37, 42, 55). On ^18^F-FDG-PET/CT diffuse tracer uptake might be seen (56). After resolution of the clinical presentation, imaging might be characterized by parenchymal atrophy and loss of normal lobulations (55).

Acute kidney injury (AKI) is the most common renal toxicity in patients receiving ICI therapy (57). However, it is generally not a direct consequence of ICI’s toxicity, as it can be caused by various etiologies. Therefore, it is important to distinguish between AKI as an irAE and AKI induced by e.g. hypovolemia or acute tubular necrosis (57). Notwithstanding, the incidence of AKI after ICI treatment is reported to be 2.2% overall and 0.6% in severe cases requiring renal transplantation (58). AKI occurs more frequently in patients treated with ICI-combination therapy (4.9%) than with anti-CTLA-4 (2%) or anti-PD-(L)1 (1.4%-1.9%) monotherapy (58). The interval between ICI treatment initiation to AKI ranges from 21 to 245 days, and from 7 to 63 days between the last ICI treatment dose and onset of AKI (58) A ccase of ipilimumab-induced immune-related kidney failure was reported with bilateral renal enlargement visualized on CT and rapid resolution after steroid therapy (59). In addition, PET/CT shows increased ^18^F-FDG uptake in the renal cortex (60, 61). Moreover, diffuse or even segmental uptake in the renal parenchyma can be seen on ^18^F-FDG PET/CT especially in delayed imaging. However, the very high pelvicalyceal activity and low spatial resolution in older generation PET/CT scanners are clear limiting factors for accurate assessment of the kidneys in patients undergoing ICI treatments (42). Therefore, for patients with clinical suspicion of AKI and contraindication for biopsies, ^18^F-FDG PET/CT might provide some diagnostic clues (60). However, the specific imaging characteristics have not been defined yet and distinguishing between immune-related and non-immune-related AKI remains challenging.

### Thoracic toxicities

**Figure 3:**
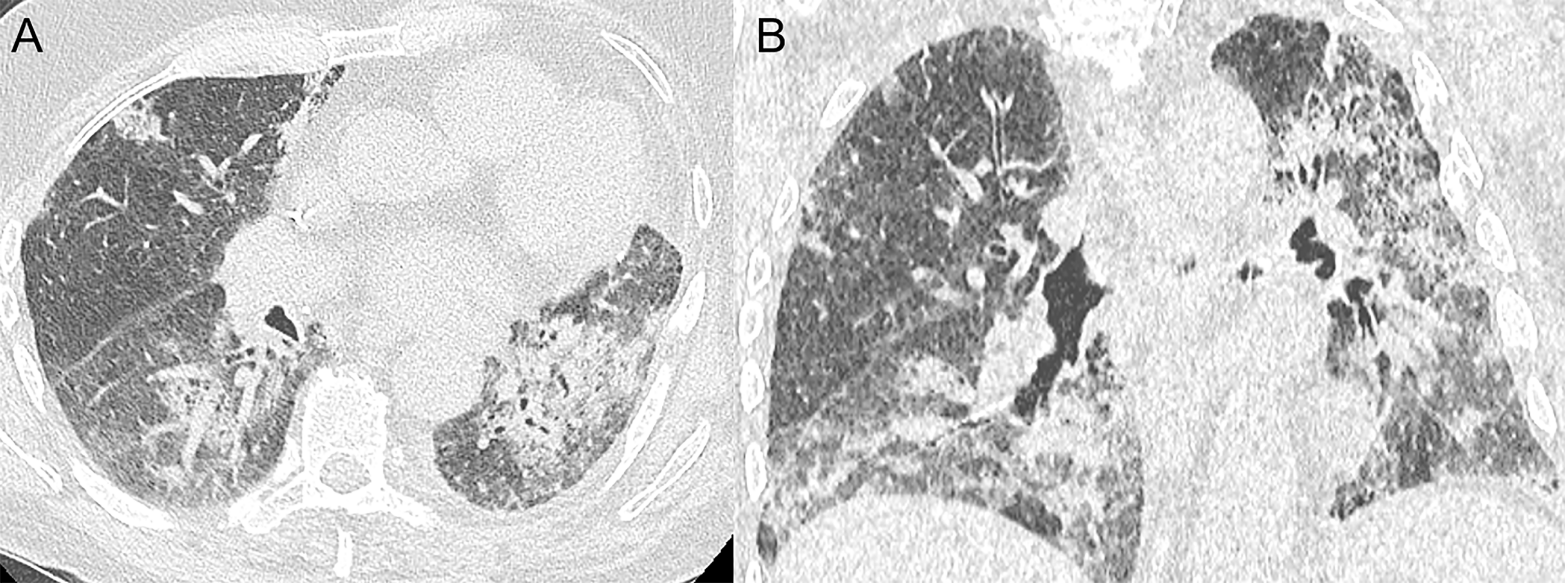
Immune-related pneumonitis in a 79-year-old male with stage IV non-small cell lung cancer in the left lower lobe. After second line treatment with nivolumab (anti-PD-1) (5 cycles), the patient developed progressive dyspnea and dry cough. Axial **(A)** and coronal **(B)** CT images demonstrate multifocal alveolar consolidations in a predominantly peribronchovascular and subpleural location compatible with a drug-induced pneumonitis.

Pneumonitis is a relatively common irAE that manifests with clinical symptoms ranging from mild dyspnea to potential lethal respiratory failure and is associated with lower patient survival (62). Pneumonitis occurs in approximately 1% of patients receiving anti-CTLA-4 therapy and in 4% of patients receiving anti-PD-(L)1 treatment, with around 1% of the cases being severe (34). In patients receiving ICI-combination treatment (nivolumab + ipilimumab or peptide vaccines), the incidence is significantly higher at 6.6% and 1.5% for severe cases, respectively (63). The median time to onset of clinical symptoms is 4.6 months in patients receiving ICI-monotherapy versus 2.7 months in patients receiving ICI-combination therapy (64, 65). Radiologic findings of ICI-related pneumonitis range from mild interstitial abnormalities to acute interstitial pneumonia and acute respiratory distress syndrome (66). The best imaging modality in this setting is CT. Based on the CT findings, irAE-related pneumonitides can be divided into five distinct phenotypes (37, 65):

1. cryptogenic organizing pneumonia-like pneumonitis with patchy or confluent consolidation with or without air bronchograms and predominantly peripheral or subpleural distribution (**Figure 3**),
2. ground glass opacities with variable expression and location,
3. increased interstitial markings, interlobular septal thickening with peribronchovascular infiltration,
4. hypersensitivity with centrilobular nodules, bronchiolitis-like appearance, and tree-in-bud micronodularity, and
5. lesions which cannot be further classified.

Ground glass opacities (55%) and consolidations (32%) non-segmentally distributed in the dominant lung or bilaterally opposite the tumor have been shown the most frequently in ICI-treated non-small cell lung cancer patients according to a systematic review by Zhang et al. (67).

A case of progressive pleural effusion as rare irAE has been reported for instance in a non-small cell lung cancer patient treated with cisplatin, pemetrexed, and pembrolizumab (68) and as late toxicity in a primary lung adenocarcinoma patient following 94 cycles of nivolumab (69). Importantly, CT scans are also of interest for ruling out differential diagnoses such as pulmonary embolism.

On PET/CT an interstitial pneumonia type pattern characterized by non-specific moderate to intense ^18^F-FDG uptake might be seen (70). However, one major diagnostic challenge is to distinguish infectious diseases from tumor lesions, e.g., nodular aspects that mimic tumor recurrence, whereas an underlying disease, such as chronic obstructive pulmonary disease, may further complicate the final diagnosis (71).

In terms of clinical management, corticosteroids are recommended as primary therapy approach based on severeness of the case and clinical expertise (67). In addition, for patients with grade 3-4 ICI-induced pneumonitis, ICI-treatment should be discontinued immediately and permanently. Clinical improvement, especially in low-grade disease is usually observed within 48-72 hours of corticosteroid use. Patients with grade 2 pneumonitis, who resolved symptoms show the highest overall survival (86%) compared with grade 3 or 4 pneumonitis (36% or 43%, respectively) (67).

**Figure 4:**
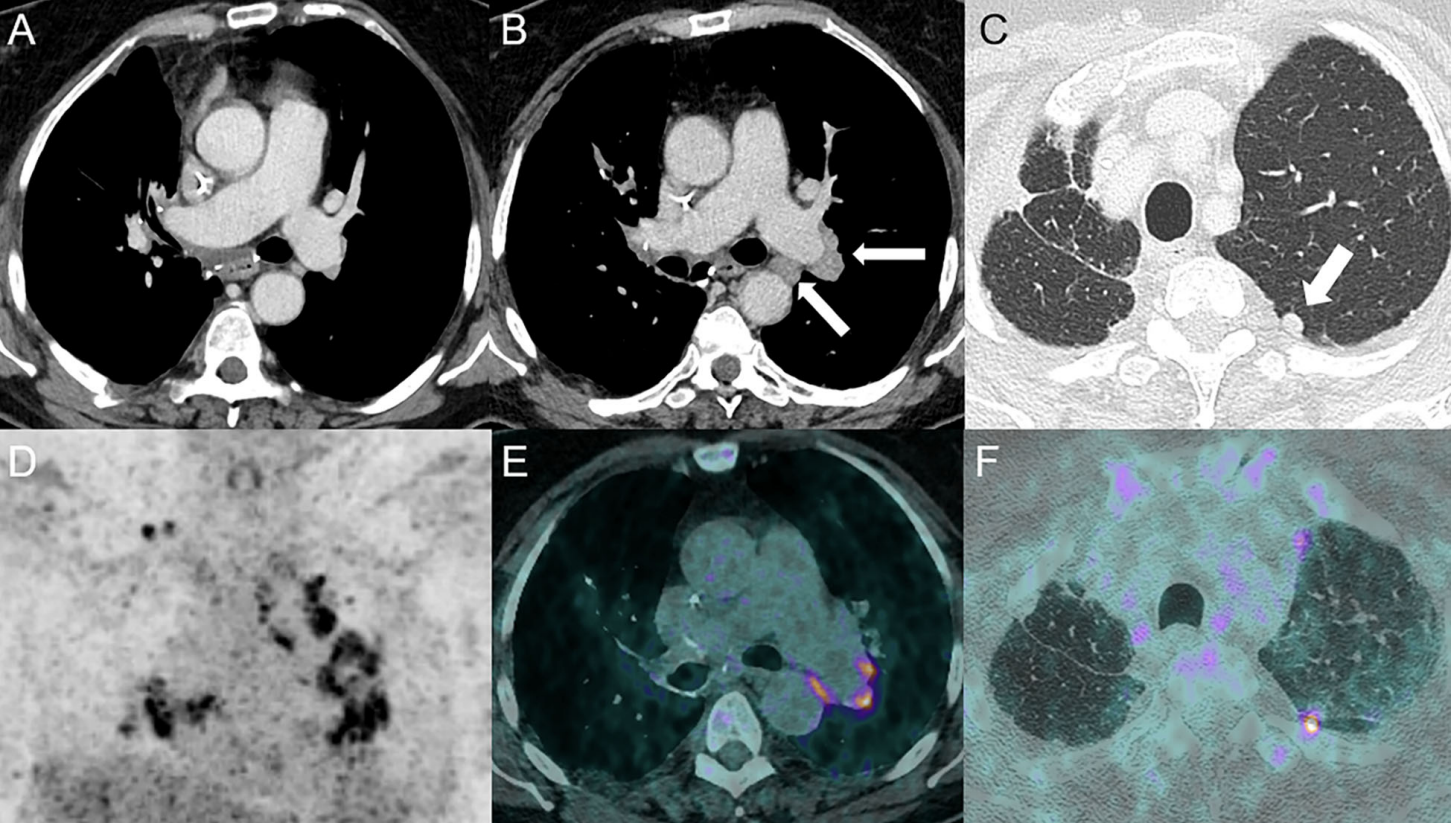
Sarcoidosis-like reaction in a 69-year-old female with stage IIIa lung adenocarcinoma in the right upper lobe treated by neoadjuvant cisplatin-docetaxel followed by durvalumab (anti-PD-L1) with subsequent right upper lobe lobectomy and lymphadenectomy. The patient received adjuvant durvalumab 1 month post-surgery. Baseline CT following surgery is shown in **(A)**. Follow-up CT at 5 months showed the development of bilateral hilar and mediastinal lymphadenopathies [arrows, **(B)**] and an increasing nodule in the left upper lobe (arrow) **(C)**. ^18^F-FDG PET-CT confirmed high uptake of mediastinal and hilar lymph nodes [PET **(D)** and fused PET/CT **(E)** images] and the upper left lobe nodule [fused PET/CT images **(F)**]. A wedge resection confirmed the sarcoidosis-like nature of the nodule.

Immunotherapy-related sarcoid-like reactions are often asymptomatic and appear in 5–7% of patients (37, 72). They might be related to the involvement of primary and secondary systemic lymphoid organs in the systemic antitumor response required for effective ICI treatment (37, 72, 73). In general, the formation of sarcoid-like granulomas occurs most frequently in lymph nodes (71%), lungs (60%), and skin (55%) and can be easily confused with disease progression or tumor recurrence (7, 74). The median time between initiation of ICI treatment and the development of sarcoid-like reactions is 14 weeks (37, 75). During the course of ICI therapy, metabolic changes in lymphoid organs could be monitored using ^18^F-FDG-PET/CT. Indeed, an increase in immune cells populations and their higher growth rate leads to higher energetic requirements often translating in high avidity for ^18^F-FDG (76, 77). Imaging findings include a new bilateral symmetric mediastinal and hilar lymphadenopathy resembling sarcoidosis in up to 10% of the patients (78), often high ^18^F-FDG avidity on PET/CT, and an association with (subpleural) perilymphatic distribution of micronodules without suspicion of intercurrent infection or new metastasis (**Figure 4**) (37, 79). It is critical to recognize these sarcoid-like irAEs as a classic response pattern to immunotherapy (initial increase of the tumor burden unconfirmed at the next imaging follow-up) in order to distinguish it from true progression or pseudoprogression (80). In cases where the diagnosis on imaging is unclear, an assessment of angiotensin converting enzyme serum levels can be performed, as elevated levels have been associated with ICI-induced sarcoidosis-like reactions (75). If true tumor progression is still suspected after this, a targeted biopsy should be strongly considered for definitive diagnosis (75).

**Figure 5:**
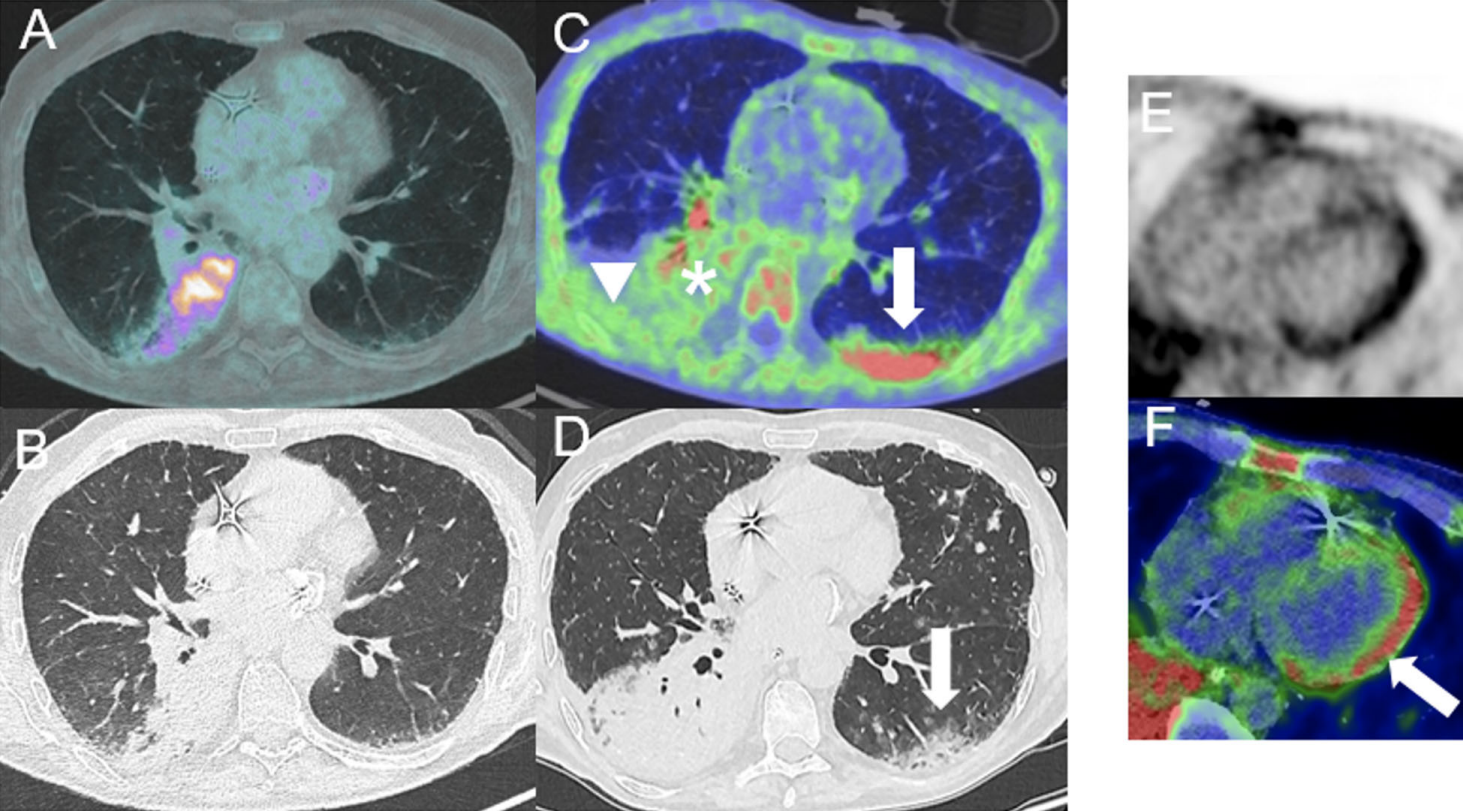
Immune-related myocarditis in a 61-year-old male with mucinous lung adenocarcinoma in right lower lobe [cT4 (>7cm) cN0 cM0] initially treated with carboplatin, vinorelbine and radiation therapy followed by consolidation treatment with durvalumab (anti-PD-L1). Baseline (before ICI) fused axial ^18^FDG PET/CT image **(A)** and corresponding axial CT image **(B)**. After 2 cycles of durvalumab, the patient experienced severe dyspnea, atrial fibrillation leading to cardiogenic shock with clinical suspicion of ICI-related myocarditis. ^68^Ga-DOTATOC-PET/CT showed necrotic areas in the lung cancer [asterisk, **(C)**] with presence of peripheral inflammatory/infectious uptake [arrowhead, **(C)**] and newly appeared subpleural alveolar consolidations in the left lower lobe compatible with an organizing pneumonia [arrow, (**C, D)**]. ^68^Ga-DOTATOC-PET/CT showed diffuse myocardial uptake in the left ventricle (LV) [**(E, F)**], with an increased uptake ratio of 2.6 (SUVpeak LV_myocardium_/SUVmean LV_cavity_) suggestive of myocarditis [arrow, fused axial PET/CT image **(F)**].

Cardiac toxicities associated with ICI treatment are relatively rare. Myocarditis, as the most common one, occurs in 0.1% to 1% of patients with symptoms such as dyspnea (49%), weakness (25%), chest pain (17%), syncope (9%), fever (6%), and cough (4%) (20, 21, 81). In most of these cases, the onset is shortly after initiation of the ICI therapy, and because of the high mortality rate of 50%, it is of the utmost importance to make the diagnosis and start the appropriate treatment as early as possible (20, 21, 82). Besides clinical features, laboratory markers and electrocardiogram changes, non-invasive imaging modalities, especially cardiac MRI (CMRI) has become more and more important in the diagnostic workup, to reduce the necessity of invasive biopsies as the current diagnostic gold standard (83, 84). In clinical practice, transthoracic echocardiography (TTE) is the first imaging modality that should be performed if acute myocarditis is suspected. Suggestive TTE findings include abnormalities of the segmental wall motion, increased thickness of the left ventricular wall, global hypokinesia (fulminant myocarditis), and pericardial effusion (84). However, a recent review of 88 ICI-induced myocarditis cases showed normal morphological TEE findings in 23% and normal left ventricular ejection fraction in 32.5% (81). Regarding CMRI, at least one criterion on T2-based (regional or global increase in myocardial relaxation time or increased signal intensity) with at least one criterion on T1-weighted imaging (increase in myocardial T1, extracellular volume, late gadolinium enhancement) should be analyzed for sufficient diagnostic accuracy according to the recently updated Lake Louise criteria (85). In 48% of cases late gadolinium enhancement (LGE) predominantly distributed in the anteroseptal, inferoseptal, inferior, and inferolateral segments (atypical localizations possible), and in 28% of the cases myocardial oedema in T2-weighted short-tau inversion recovery (STIR) is described (83, 86). However, these characteristics are limited due to their low specificity. In a study of an international registry of patients with ICI-associated myocarditis (n=103), only 48% of patients with ICI-induced myocarditis had LGE when compared to 90% of patients with other causes of myocarditis (83). Herby it is important to note that CMRI assessment >4 days after admission showed significantly more positive LGE findings than if CMRI was performed earlier (72.0% vs 21.6%, p<0.001) (83). Moreover, LGE was not associated with clinical symptoms, patient outcomes, ECG or echocardiographic findings. Finally, nuclear medicine findings might provide important clues for the diagnostic of immune-related acute myocarditis (**Figure 5**). Interestingly, ^18^F-FDG-PET/CT has a very limited role in this setting as demonstrated in a recent study of 61 patients with suspicion of ICI-related myocarditis where its sensitivity was below 30% (87). However, other PET tracers have been proven useful in this context, such as ^68^Ga-DOTATOC which showed high sensitivity for the early detection of pathological myocardial uptake in a small population of patients (n=9) with clinical suspicion of ICI-related myocarditis (88). A pathological diffuse tracer uptake in the myocardium was the most frequent pattern detected. Interestingly, ^68^Ga-DOTATOC PET/CT showed a good correlation with elevated serum cardiac troponin I and immune correlates such as inflammatory cytokines (IL-6) and chemokines (CXCL9, CXCL10 and CXCL13) by contrast evocative lesions for myocarditis were only seen in 3 out of the 8 patients that had a CMRI (88).

Pericarditis is reported to be the second most common immune-related cardiotoxicity, although data is lacking regarding its exact incidence (82). The median onset of pericardial disease is estimated to be 30 days (82). Symptoms include shortness of breath, pericardial pain without pericardial effusion or jugular vein congestion, and cardiogenic shock with cardiac tamponade due to pericardial effusion, resulting in a high mortality rate of 21% (82, 89). Moreover, pericardial toxicity can occur alone or in combination with ICI-associated myocarditis (myopericarditis) (89). The diagnostic work-up includes detailed physical examination, electrocardiogram, echocardiogram, CMRI and cardiac PET/CT (89, 90). On CMRI, ICI-related pericardial disease demonstrates focal myocardial LGE in the mid-lateral wall and mild LGE of the pericardium along the lateral wall in cases suggestive of myopericarditis (90).

### Neuromuscular toxicity

**Figure 6:**
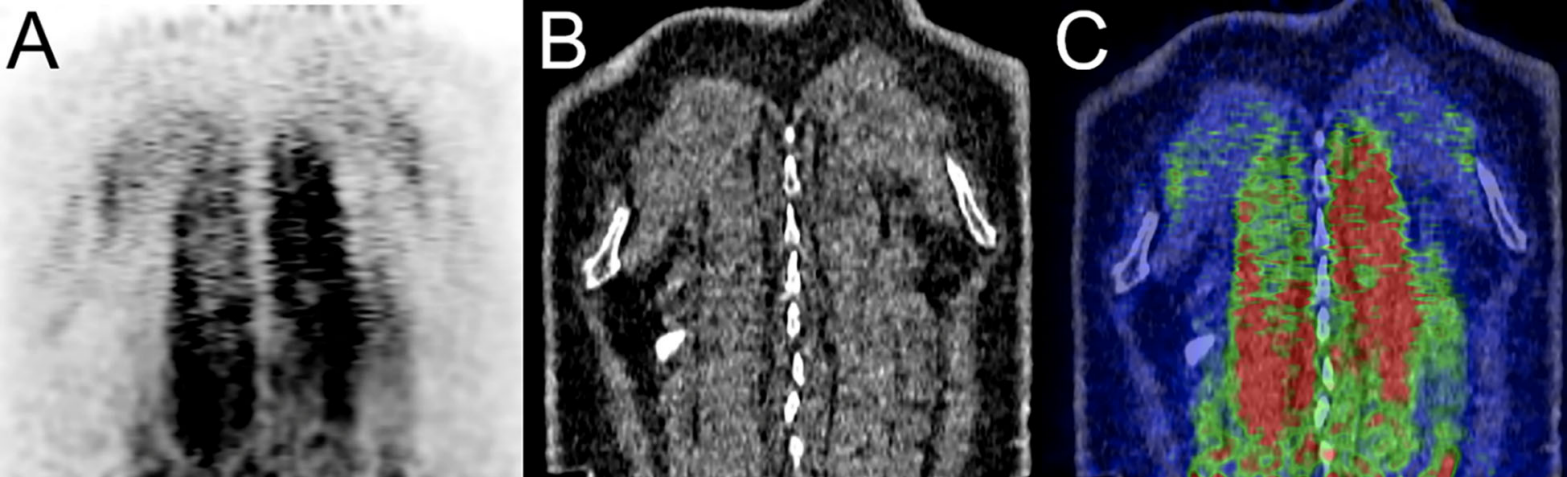
Immune-related myositis in a 61-year-old male patient with small cell neuroendocrine carcinoma of the right lung hilum (cT4 cN2 cM0, stage IIIB) initially treated with chemo-radiation therapy who developed diffuse metastatic disease. A treatment with ipilimumab (anti-CTLA-4) and nivolumab (anti-PD-1) was administered. At 1-month post-immunotherapy, a ^68^Ga-DOTATOC-PET/CT showed diffuse myositis of paraspinal muscles coronal PET, CT and fused PET/CT images **(A–C)** respectively showing spinalis, longissimus thoracis and iliocostalis thoracis muscles.

**Figure 7:**
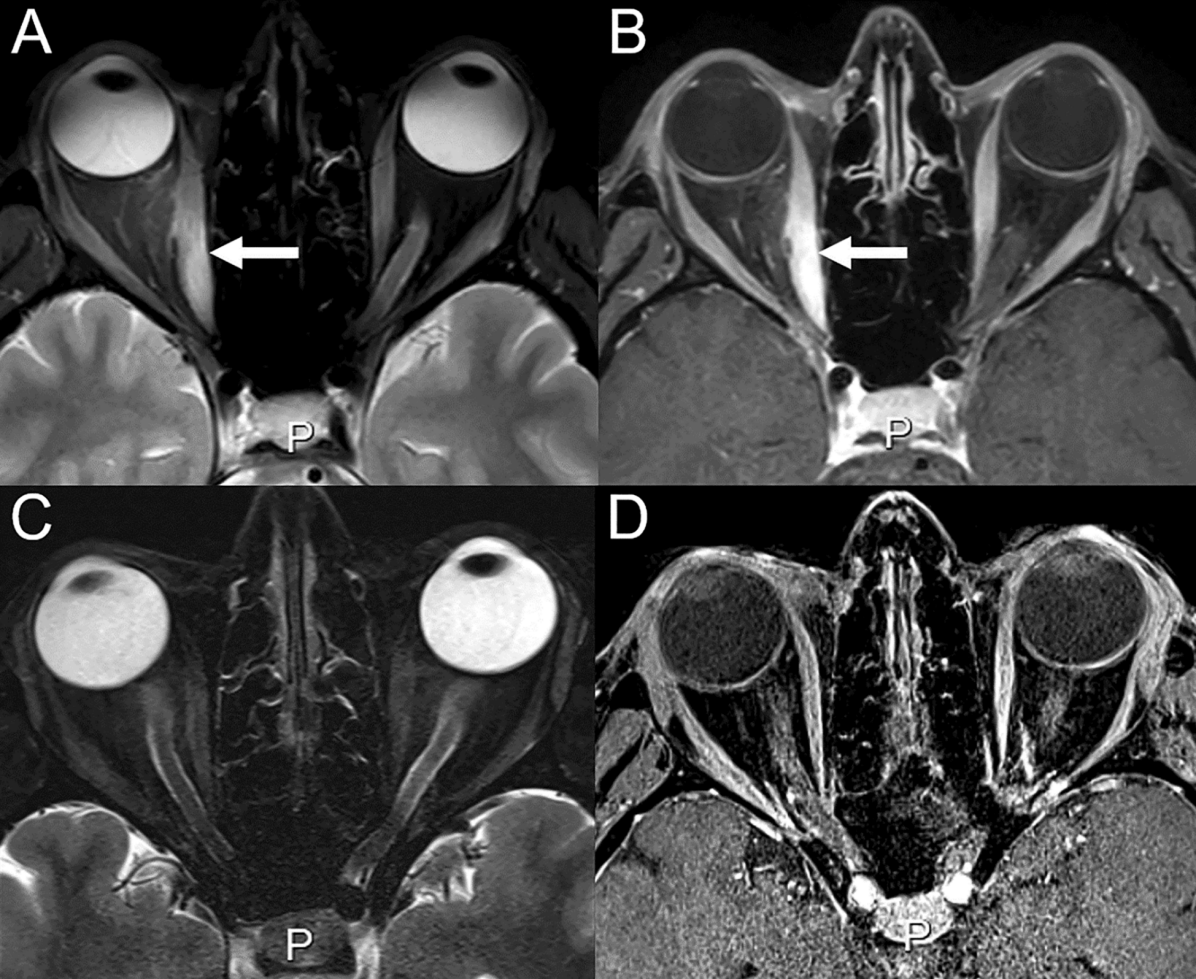
Immune-related orbital myositis in a 43-year-old female with cutaneous melanoma treated with ipilimumab (anti-CTLA-4) and nivolumab (anti-PD-1). After 3 cycles of ipilimumab and nivolumab, the patient reported diplopia. **(A)** On T2-weighted and **(B)** contrast-enhanced images, orbital edema, abnormal enhancement and thickening of the right medial occulomotor muscle can be seen, consistent with orbital myositis (arrows). **(C, D)** MRI at 1 month from treatment discontinuation with disappearance of signs of inflammation.

Regarding peripheral neuromuscular toxicities, myositis is the most common syndrome. While being the most prevalent in anti-PD(L)-1 therapy, it occurs in approximately 0.4-3% of ICI-treated patients (**Figure 6**) (91–93). The median time of ICI-administration to myositis symptom development ranges from 5 to 87 days (94). Interestingly, the clinical manifestation from immunotherapy-related myositis differs markedly from that of idiopathic and paraneoplastic inflammatory myopathies such as dermatomyositis and polymyositis. Progressive symptom development, as well as oculomotor and axial muscle involvement are uncommon, but have been reported. Bulbar symptoms, such as dyspnea, dysarthria, and dysphonia have been described (95, 96). However, sudden onset of stable myalgia with or without elevated creatine kinase is the most common symptom of immune-related myositis (96). The differential diagnosis to myastenia gravis is sometimes challenging, since on one side, myastenia gravis is often associated with optical myositis and on the other side, acetylcholine receptor binding antibodies can occasionally be detected in optical myositis in the absence of myasthenia gravis (97, 98). On brain MRI, immunotherapy-related myositis is characterized by fat-suppressed T1/T2-weighted intramuscular hyperintensity with or without gadolinium enhancement (96, 99). The ocular phenotype presents contrast-enhanced orbital edema as well as abnormal enhancement and enlargement of the extraocular muscles (**Figure 7**). Moreover, PET/CT with increased muscular ^18^F-FDG uptake can support the diagnosis and help to estimate the severity by assessing how many muscle groups are affected (96, 99). Pathological muscle uptake suggestive of myositis could also be detected using ^68^Ga-DOTATOC PET/CT as shown in 5 out of the 6 patients that presented with myositis concomitant to an ICI-related myocarditis (88). In addition, rheumatological disorders are also frequent irAEs during the course of ICI treatment and ^18^F-FDG-PET/CT could be useful for the detecting and assessing the severity of the inflammation associated with those events in particular for arthritis affecting several articulations but also tenosynovitis or polymyalgia rheumatica (41).

### Central neurologic toxicity

In contrast to peripheral nervous toxicities, irAE of the central nervous system such as encephalitis, aseptic meningitis, vasculitis, cranial neuropathies, and myelitis are uncommon (100).

Although in recent years an increasing number of immune-related encephalitis have been described and may occur with each treatment cycle, it remains a rare immune-related toxicity with an incidence of 0.1–0.2% (94, 100). These cases present a wide range of potential life-threatening symptoms, including confusion, agitation, fever, headache, fatigue, short-term memory impairment, neck stiffness, behavioral changes, and psychiatric symptoms (101, 102). The diagnostic workup usually includes brain MRI, lumbar puncture, paraneoplastic autoantibodies, electroencephalography, and laboratories, notably to rule out infectious agents related diseases. MRI, particularly T2-weighted and/or fluid-attenuated inversion recovery (FLAIR), reveals encephalitis features such as ill-defined uni- or bilateral hyperintense signals in the limbic cortex, the cerebellum, basal ganglia or scattered in the gray or white matter, with or without enhancement corresponding to zones of inflammatory infiltrates and epileptogenic activity (93, 103, 104), some being associated with auto-antibodies (105). Multifocal lesions involving the white matter, optical nerve, and spinal cord, which mimic demyelinating diseases, have also been described (106, 107). The physiological high ^18^F-FDG uptake of the brain limits somehow the irAEs assessment using ^18^F-FDG-PET/CT (41). However, there is evidence of the utility of ^18^F-FDG-PET/CT, showing increased or decreased metabolic activity, to detect ICI-induced encephalitis earlier than standard diagnostic approaches (108). A recent study in patients with autoimmune encephalitis, which shares many similarities with ICI-related encephalitis, described 6 cases with metabolic abnormalities on ^18^F-FDG-PET/CT with normal MRI (n=2), lumbar puncture (n=3), and electroencephalography (n=2) findings (109). Finally, cases of posterior reversible encephalopathy syndrome have been occasionally reported following ICI administration alone or in combination with chemotherapy (110–112).

**Figure 8:**
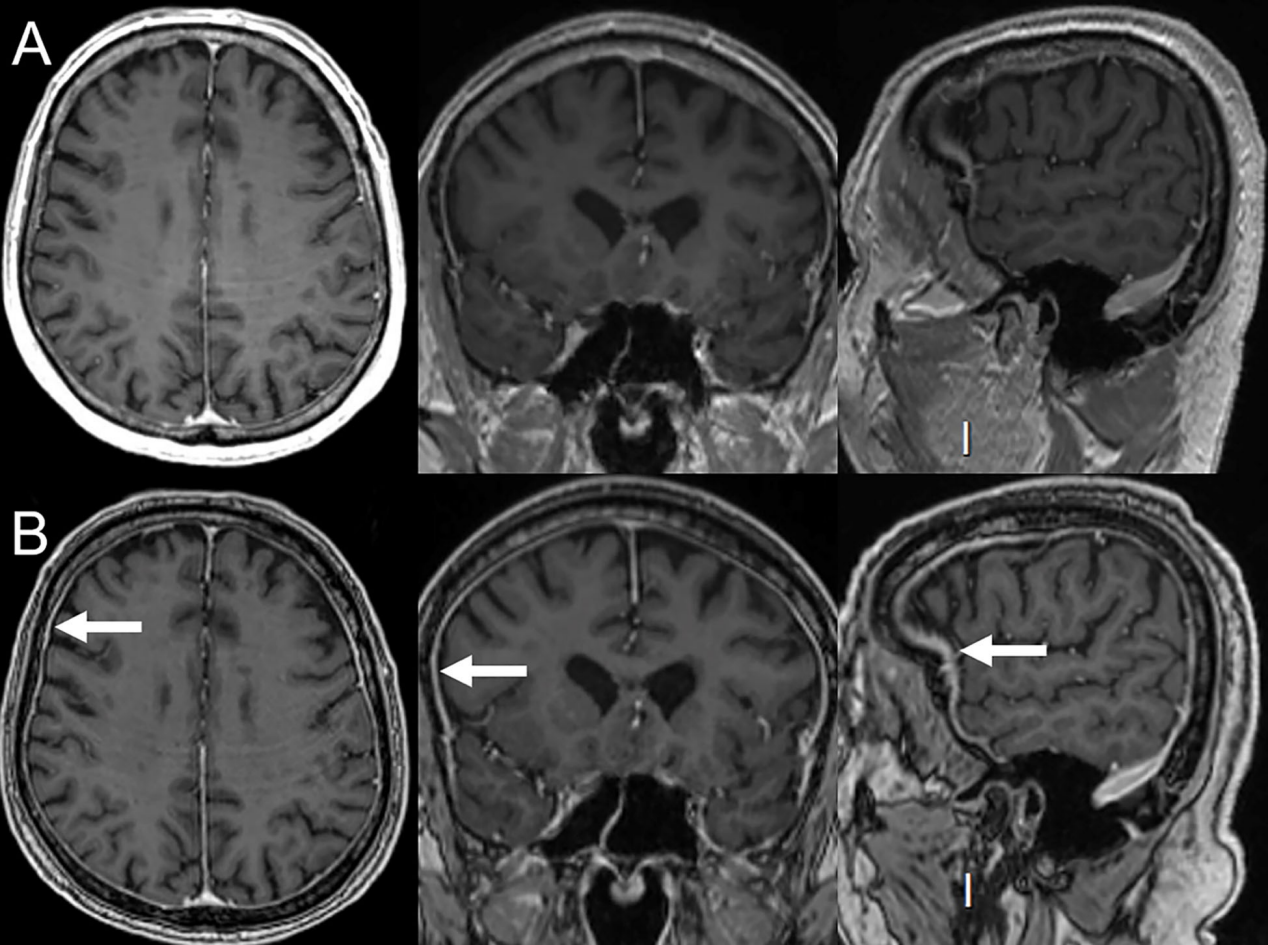
Immune-related aseptic meningitis in a 78-year-old female with NSCLC treated with ipilimumab (anti-CTLA-4) and nivolumab (anti-PD-1). **(A)** Normal brain MRI (axial, coronal and sagittal plans) performed 6 months prior to immunotherapy. After 3 cycles of ipilimumab and nivolumab, the patient developed headaches. **(B)** MRI performed 2 months after the beginning of immunotherapy showed smooth diffuse dura mater thickening (arrows) compatible with aseptical meningitis. Patient’s symptoms and signs of inflammation on MRI disappeared upon immunotherapy discontinuation.

Aseptic meningitis is present in <0.1% of ICI-treated patients overall (93). This condition is more commonly associated with anti-CTLA-4 and ICI-combination treatments (93, 100). Moreover, the time to clinical disease onset is short, with a delay of 9 days from the first dose of immunotherapy (100). Aseptic meningitis is characterized by the subacute onset of nonspecific symptoms such as headache, neck stiffness, photophobia, low-grade fever, and nausea, and must be distinguished from infectious or carcinomatous causes of meningitis (93). In 42% of the patients, brain MRI shows diffuse leptomeningeal enhancement with or without parenchymal abnormalities as a nonspecific sign of inflammation and is consistent with the presence of lymphocytic or neutrophil pleocytosis, while overlapping with findings of immune-induced meningoencephalitis (113, 114)(**Figure 8**). However, 46% of brain MRI findings are normal in ICI-induced aseptic meningitis (113). However, this even underlines the importance of imaging to rule out differential diagnosis such as (ischemic) stroke, infection, and brain metastasis.

In recent years, there is increasing evidence for ICI-associated central nervous system vasculitis. However, the exact frequency (currently estimated to be <0.01%), timing and association with a specific type of immunotherapy is still unclear (93). Commonly reported types of vasculitis are large vessel vasculitis (giant cell arteritis and isolated aortitis) and vasculitis of the nervous system (primary angiitis of the central nervous system, PANCS, and isolated vasculitis of the peripheral nervous system) (93, 115). With a median time of 3 months from the initiation of ICI treatment to their development, symptoms are often unspecific and include headaches (60%), altered cognitive status (50%), and focal neurologic deficits. Most commonly, they are mild, and no fatalities related to vasculitis have been observed (93, 115). Although considered to be the gold standard for diagnosis, biopsy of the brain and/or spinal cord showing segmental inflammatory infiltration leading to blood vessel walls thickening and stenosis, resulting in decreased blood flow or even secondary to hemorrhagic vessel rupture, has only a sensitivity of 53% (116). However, this sensitivity can be increased to more than 80% by identifying focal lesions previously on neurologic imaging techniques (117). Brain MRI is altered in more than 90% of patients with PANCS, showing (nonspecific) signs of microangiopathy, hemorrhage, or ischemic infarction, as well as multifocal bilateral T2- weighted, FLAIR and diffusion-weighted sequence abnormalities in the cortical-subcortical area (118). However, the occasional presence of solid lesions and gadolinium enhancement of leptomeninges complicate the distinction to tumors and abscesses and requires additional imaging modalities such as CT angiography, high-resolution contrast-enhanced MRI, or ^18^F-FDG-PET/CT to detect vascular inflammatory activity (119).

### Endocrine toxicities

Endocrinopathies are observed in up to 10% of patients treated with anti-CTLA-4 and in 4-14% of patients treated with anti-PD-1 therapy (120, 121).

**Figure 9:**
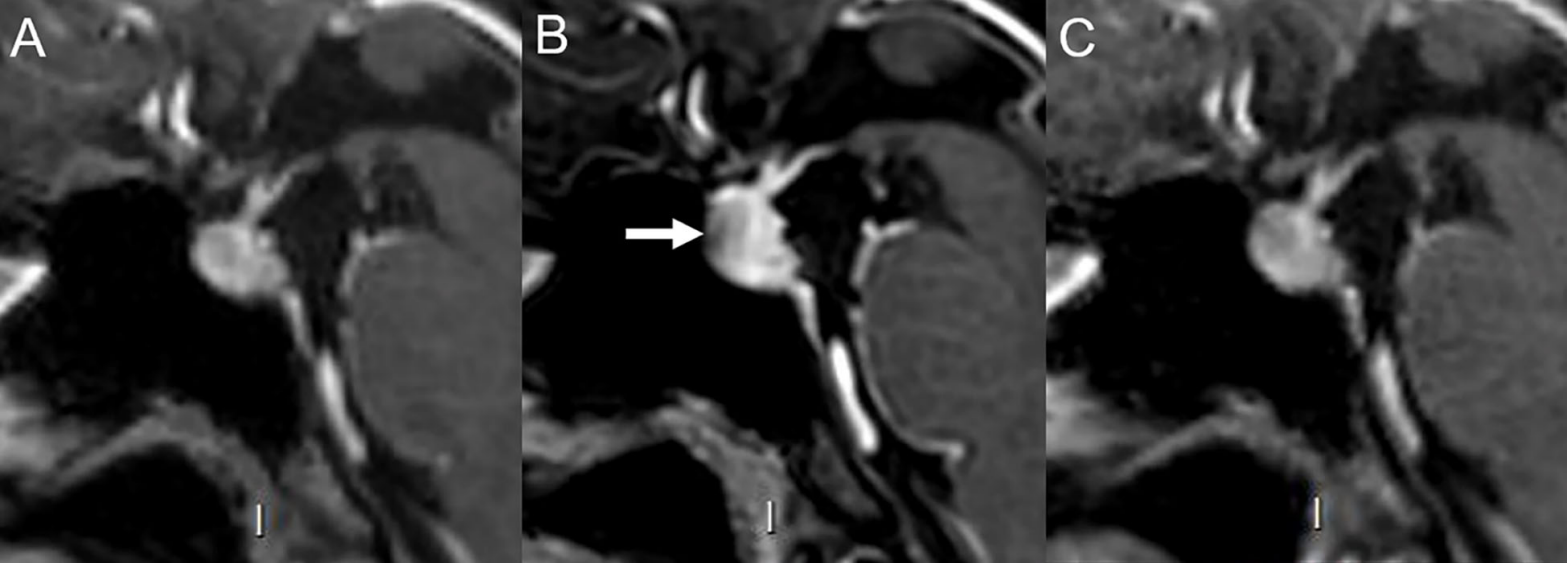
Immune-related hypophysitis in a 43-year-old female with cutaneous melanoma treated with ipilimumab and nivolumab. **(A)** Normal hypophyseal MRI performed 4 months prior to immunotherapy. After 3 cycles of ipilimumab (anti-CTLA-4) and nivolumab (anti-PD-1), the patient developed headaches. **(B)** MRI revealed increased hypophyseal height with mild pituitary stalk thickening and reduced opto-chiasmatic cistern size compatible with hypophysitis (arrow). Ipilimumab was discontinued and 2 more cycles of nivolumab alone were administered. Eventually, nivolumab was discontinued due to a grade 3 toxidermia. **(C)** Hypophyseal MRI performed 3 months after the last immunotherapy cycle was normal with disappearance of signs of inflammation.

Hypophysitis occurs in 4.5% (0.8% severe cases) of the patients treated with anti-CTLA-4, whereas it is reported in less than 1% (0.1%) of patients with anti-PD-(L)1 treatment (122). The clinical features of pituitary dysfunction can be nonspecific and include fatigue, headache, or weakness with additional symptoms related to hypopituitarism (122, 123). The median time to symptom onset ranges between 11 weeks (ipilimumab), 17 weeks (combination of impilimumab and nivolumab), 22 weeks (nivolumab), and 26 weeks (pembrolizumab) (124). Since pituitary inflammation can be caused by ICI therapy as well as by pituitary metastasis and adenomas, MRI and ^18^F-FDG-PET/CT are playing a crucial role in distinguishing these diseases as they often show imaging findings of immune-related hypophysitis before the appearance of symptoms (18, 42, 45). Contrast-enhanced MRI of immune-related hypophysitis shows enhancement of the posterior portion of the pituitary gland in 89% of the patients, whereas the enhancement is homogeneous in 63.3% (vs. heterogeneous enhancement, 36.7%) (16, 125) (**Figure 9**). This pattern is important for distinguishing this toxicity from pituitary metastasis, as differential diagnosis, which show heterogeneous enhancement in the vast majority of cases (82.6%) (16). Moreover, thickening of the pituitary stalk has been identified in 29/49 (59.2%) cases of hypophysitis and only in 16/58 (27.6%) cases with pituitary metastasis (16). On PET/CT, immune-related hypophysitis shows ^18^F-FDG-avid pituitary gland often enlarged but without mass effect on the optic chiasm and with thickening of the infundibulum (125, 126). A recent study in 162 advanced melanoma patients who received ipilimumab/nivolumab combination therapy showed that ^18^F-FDG-PET/CT was able to predict the appearance of hypophysitis with high positive (86%) and negative (87%) predictive values (127).

**Figure 10:**
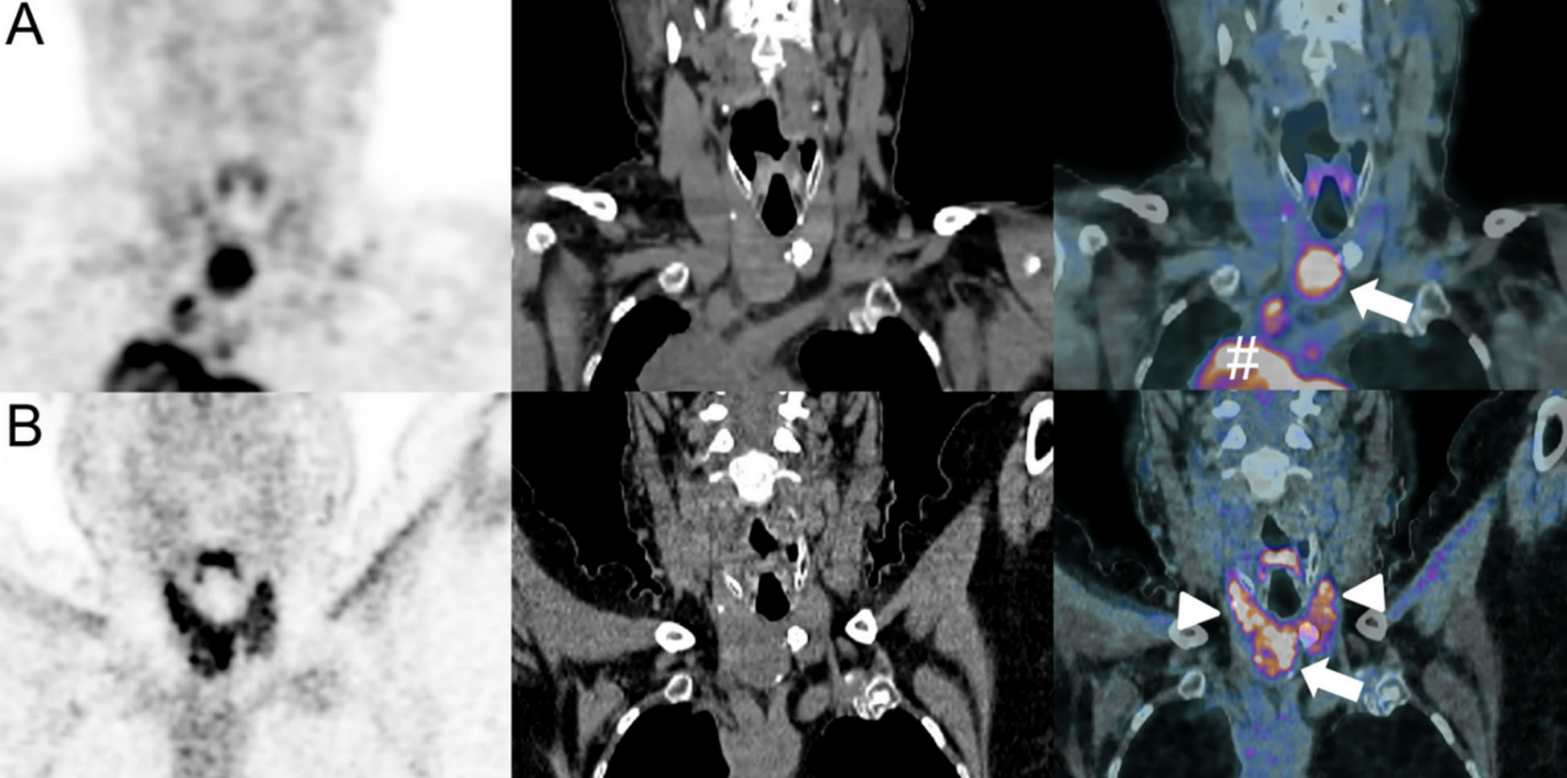
Immune-related thyroiditis in a 50-year-old female with lower leg Meckel-cell carcinoma who developed mediastinal metastatic spread as shown on baseline ^18^F-FDG-PET/CT [coronal PET, CT and merged PET/CT images, # **(A)**]. A thyroid nodule was also present [arrow, fused PET/CT image **(A)**]. Pembrolizumab (anti-PD-1) was administered. Follow-up 18F-FDG-PET/CT at 6 months showed disappearance of mediastinal disease and decrease in metabolic activity of the thyroid nodule [arrow, fused PET/CT image **(B)**]. However, a marked increase in thyroid activity was also evident, consistent with a thyroiditis [arrowheads, fused PET/CT image **(B)**].

ICI-induced thyroid dysfunction is often clinically asymptomatic and transient, and identified by blood tests as mild hypo- or hyperthyroidism associated with elevated anti-thyroid peroxidase and/or anti-thyroglobulin antibodies (128). In terms of frequency, hypothyroidism is more common, affecting 15% of patients receiving ICI-combination therapy, 3% of patients receiving anti-CTLA-4, and 8% of patients receiving anti-PD-(L)1 therapy (34). Hyperthyroidism is observed in only 4% of patients treated with anti-CTLA-4 and 5% of patients treated with anti-PD-(L)1 molecules (34). In few cases, ICI therapy did lead to Graves’ disease (129). Immune-related thyroiditis, which usually occurs within 5 to 10 weeks following treatment, is mostly mild and CTCAE grade ≥3 is rarely observed (34, 130). US is the imaging modality of choice and new enlargement of the thyroid gland with heterogeneous hypoechoic parenchyma, (pseudo)nodular pattern, and increased vascularity on color Doppler is commonly observed (37, 42). CT findings are unspecific as they present a new enlargement of the thyroid gland associated with a heterogeneous parenchymal enhancement (37, 42). Still, thyroiditis remains frequently an incidental finding on ^18^F-FDG-PET/CT with a diffuse increased uptake of the thyroid gland (**Figure 10**) (37, 42).

Compared with the more common secondary adrenal insufficiency caused by pituitary dysfunction, primary adrenal insufficiency, in which the adrenal glands are directly damaged due to ICI therapy, has been rarely described (131). Adrenal insufficiency is estimated to occur in 5% of patients treated with ICI-combination therapy and in 1% of patients treated with anti-CTLA-4 or anti-PD-(L)1 mAbs, whereas CTCAE grade ≥3 is rarely reported (34, 132). Disease onset after initiation of ICI treatment is in between 9 weeks (ipilimumab), 3.3 month (pembrolizumab) and 5 months (nivolumab) (121, 133). Symptoms are characterized by electrolyte abnormalities, dehydration and altered mental status. Life-threatening adrenal crisis with vasodilatator shock and hypotension, requiring permanent steroid replacement therapy, was reported following nivolumab therapy (134). Therefore, rapid diagnosis and close monitoring are required. On CT and MRI, adrenal glands show bilateral, symmetrical, and smooth enlargement, while uniform mild hypermetabolism is seen on ^18^F-FDG PET/CT (42, 135).

## Current challenges and future directions

The increasing use of immunotherapies in clinical practice has led to the challenge of individually managing their treatment-related toxicities. It is particularly important to distinguish patients who benefit from therapy from those who are at risk of experiencing drug-related toxicities. Imaging plays a central role in the detection and characterization of these irAEs as well as in the differentiation of immunotherapy-associated response patterns such as pseudoprogression . In severe cases, appropriate treatment of these ICI-induced toxicities must be initiated as early as possible, and it may even be necessary to discontinue ICI treatment. However, it is important to note that many ICI-induced toxicities are mild and manageable. Since the increased use of imaging may lead to important financial costs and resources associated with e.g. monitoring of these irAEs with imaging, it is crucial to define parameters, to distinguish patients who benefit from imaging follow-up from patients for whom blood-based monitoring or simply clinical monitoring is sufficient. Moreover, the role of imaging still needs to be defined in other ICI-related phenomena, such as the presumably rare and previously poorly described but possibly fatal cytokine release syndrome which occurs usually within 4 weeks of ICI-treatment initiation (136, 137).

Interestingly, the occurrence of (low-grade) irAEs has been correlated with treatment efficacy and improved clinical outcomes as measured by overall response rate, progression-free survival and overall survival (6, 17). Furthermore early-onset immune-related hepatitis as irAEs was used to detect pseudoprogression and to distinguish this response pattern from true progression in a case of metastatic ovarian cancer treated with nivolumab (138).

A current research topic is the use of radiomics and deep learning techniques to evaluate and even predict cancer therapy success. Radiomics has already been proven to predict toxicity in the assessment of chemotherapy (139). Liver toxicity could be identified using liver texture analysis on the first follow-up CT before any increase in liver function tests could be detected in a proof-of-concept study of colorectal cancer patients treated with 5-fluorouracil (139). It is conceivable that similar approaches can be used to identify patients who benefit most from immunotherapies, as opposed to patients at higher risk for developing irAEs (140, 141). Preliminary studies in non-small cell lung cancer patients showed promising results. Radiomics could potentially predict the development of ICI-induced pneumonitis based on baseline CT characteristics with 100% accuracy (*p* = 0.0033) and a strong predictive power (area under the curve 1.0, *p* = 0.0033) (142). Despite the limited size of the training sample (2 patients who developed pneumonitis and 30 patients who did not), these results may help to stratify patients at risk for developing pulmonary toxicities and therefore allowing for pre-treatment modifications and changes of the therapy. Moreover, radiomic signatures on baseline CT have been shown to be more sensitive than clinical findings in identifying patients at risk for developing ICI-induced pneumonitis (143). Furthermore, radiomic features extracted from ^18^F-FDG PET/CT might provide important clues for the prediction of irAEs. A retrospective study of 146 patients with advanced non-small cell lung cancer was used to develop a multi-factorial radiomic model based on a radiomic score, generated using features extracted from PET, CT and PET/CT fusion images of baseline ^18^F-FDG-PET/CT (117). The combination of high radiomics score values with the type and dose of immunotherapy have been shown to be associated with the development of severe irAE (144). These findings underscore the value of a comprehensive baseline imaging analysis in patients treated with ICIs, as it could help predicting and preventing even life-threatening irAEs that may not be detected during baseline clinical or biological assessments.

Recently, several studies have demonstrated an association between irAEs detected on ^18^F-FDG PET/CT and favorable clinical outcomes, suggesting the value of ^18^F-FDG PET/CT in predicting responses to immunotherapy (32, 78, 145, 146). In 10% of patients with unresectable metastatic melanoma treated with ipilimumab who underwent interim or late ^18^F-FDG-PET/CT sarcoid-like mediastinal-hilar lymphadenopathy was reported and all these patients showed disease control (78). This pattern was not seen in patients with progressive disease, suggesting an association of sarcoid-like reactions with clinical benefits of anti-CTLA-4 therapy. Similarly, a small study of 16 patients with BRAF-mutated metastatic melanoma treated with vemurafenib/ipilimumab combination therapy who underwent ^18^F-FDG-PET/CT detected 7 patients developing at least one irAE (most frequently colitis and arthritis) (146). All these patients had a significantly longer progression-free survival than those without irAEs (*p =* 0.036) (146). Similary, in ICI-treated patients with either renal cell carcinoma, malignant melanoma, or lymphoma who underwent early time-point ^18^F-FDG-PET/CT, an association was found between thyroiditis and improvement of clinical symptoms at the 12-month follow-up (32). This finding was confirmed in another study which examined 91 patients treated with anti-PD-L1 therapy, suggesting that immune-related thyroiditis could be a potential predictor of response to ICI treatment (147). Overall, although imaging such as ^18^F-FDG PET/CT can contribute to the (early) detection of irAEs and irAE detected on this imaging modality might contribute to predict patient’s prognosis, these findings must always be considered in the context of patient’s symptoms (if any), comorbidities, and other findings (e.g., laboratory values) in order to decide whether or not ICI treatment should be continued.

The recent development of immune-PET tracers may improve ICI response monitoring and diagnosis of irAEs by increasing the specificity of pathological uptake seen on molecular imaging compared with ^18^F-FDG-PET/CT which, although widely used, has poor cell specificity (76, 148). This could be particularly useful when findings on ^18^F-FDG-PET/CT are inconclusive and cannot distinguish between irAEs and true or pseudo-progression. In this setting, ^89^Zr and ^64^Cu-Keytruda could be useful as anti-PD-1 human antibody immuo-PET tracers as they represent a specific imaging modality for PD-1-expressing tumor-infiltrating lymphocytes (149). Similarly, ^89^Zr-Nivolumab uptake on PET/CT correlates with PD-1-expressing lymphocytes and offers the possibility of a real-time imaging of tumor infiltrating T-cells (150). Granzyme B-targeted PET tracer (GZP) as another novel PET tracer for detection of irAEs, has also shown promising results in a murine model (151). This recent study showed an increased uptake of GZP in organs affected by irAEs and a decreased uptake after anti-inflammatory treatment, with a good correlation with immune infiltration on histology (151). This is all the more interesting since granzyme B was also found in colon and kidney samples of patients with irAEs, suggesting its potential utility in routine practice for patients treated with ICI (151, 152). However, even though novel immune-PET tracers seem to be useful and to provide important clues mainly in nonspecific cases, most findings are based on small (preclinical) studies.

**Figure 11:**
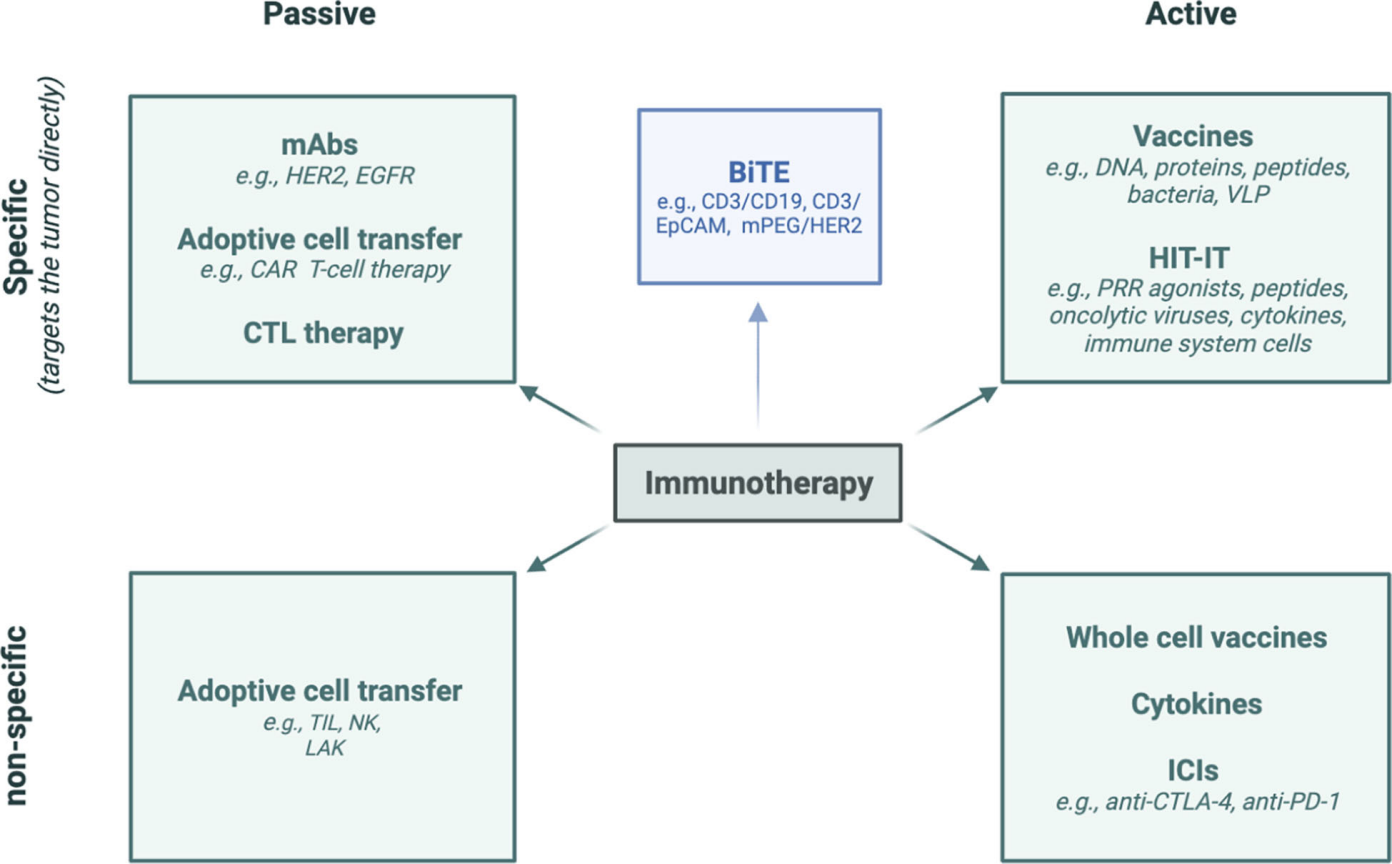
Diverse methods of immunotherapy. HER2, human epidermal growth factor receptor 2; EGFR, Epidermal Growth Factor Receptor; CAR, chimeric antigen receptor; CTL, cytotoxic T-lymphocyte; TIL, tumor-infiltrating lymphocyte; NK cell, natural killer cell; LAK cell, lymphokine-activated killer cell; BiTE, Bi-specific T-cell engagers; EpCAM, epithelial cell adhesion molecule; DNA, deoxyribonucleic acid; VLP, virus-like particle; HIT-IT, human intratumoral immunotherapy; PRR: pattern-recognition receptor; ICI, immune checkpoint inhibitor; CTLA-4, cytotoxic T-lymphocyte-associated protein-4; PD-1, programmed cell death protein-1; PD-L1, programmed death-ligand 1.

In addition to ICI therapy, there are many other types of immunotherapies that may be associated with different spectra of irAEs (**Figure 11**). Chimeric antigen receptor (CAR) T-cell therapy has been shown to induce rapid and durable responses in many types of cancers (153). However, treatment associated toxicities can be severe and even fatal, such as most commonly the cytokine-release syndrome which has a comparable clinical presentation to hemophagocytic lymphohistiocytosis or macrophage-activation syndrome and is characterized by hepatosplenomegaly, hepatic dysfunction, hyperferritinemia, hypofibrinogenemia, and coagulopathy (153, 154). In addition, irAEs associated with CAR T-cell therapy include the immune effector cell-associated neurotoxicity syndrome, characterized by initial global aphasia (153, 154). During the course of disease, patients usually experience subclinical or clinical seizures and rarely diffuse cerebral edema within 28 days (153). The diagnostic workup includes clinical and neurological examination, an electroencephalogram, and a brain MRI (153). A single-center study investigated 133 patients with relapsed and/or refractory CD19^+^ B-cell acute lymphatic leucemia, non-Hodgkin lymphoma, and chronic lymphoid lymphoma who received CD19-CAR-T cell therapy (155). Acute abnormalities in brain MRI examinations were noted in 30% which were associated with poor outcome, especially in severe cases (155). Changes in T2-weighted/FLAIR brain MRI indicative of vasogenic edema, leptomeningeal enhancement, and/or multifocal microhemorrhages could be found in most of the patients with clinically severe neurotoxicity and abnormal MRI scans (155). In addition, contrast enhancement suggestive of blood-brain barrier breakdown has been noted in some patients (155). One patient showed extensive cortical diffusion restriction indicative of cytotoxic edema and several others showed vasogenic edema that developed into cortical laminar necrosis (155). However, larger, high-quality multicenter studies are needed to more thoroughly investigate the toxicities associated with CAR-T cells and, in particular, regarding their potentially specific imaging properties.

Interestingly, peptide-based vaccines show a better tolerance and safety compared with conventional chemotherapy and ICI, and serious irAEs, such as pulmonary embolism, are rarely described (156). A meta-analysis that included 500 patients demonstrated that only 1.2% of vaccinated patients suffered from serious adverse events related to the vaccine (157). The vaccine-related irAEs include most commonly erythema and induration related to the injection side (156). Moreover, nonspecific symptoms such as nausea, diarrhea, myalgia, fatigue, increased aspartate aminotransaminase and alkaline phosphatase, and rarely hematological toxicities as well as autoimmunity have been described (156).

Finally, adverse events associated with oncolytic virus therapy are also mostly mild and usually include flulike symptoms and local reactions at the injection sites (158). However, more severe toxicities such as anemia, leukopenia, lymphopenia, neutropenia, thrombocytopenia, liver dysfunction, and hematological abnormalities, pleural effusion, herpes virus infection, and central nervous system symptoms have been described (158).

Overall, no specific imaging features of irAEs have been described yet. As the clinical use of these novel treatments increases, problems in the toxicity screening will, therefore, arise. However, over time, more information on non-ICI immunotherapies will be collected that will shed light on their specific toxicity profile and help to define their imaging characteristics.

To conclude, imaging can contribute to the detection and characterization of ICI-related toxicities, while radiomics can even help to predict these toxicities. However, reliable toxicity screening of irAEs remains challenging for rarer irAEs and non-ICI immunotherapeutics. Therefore, there is a need for large-scale clinical trials across various oncologic diseases and immunotherapeutic agents to better assess the characteristics of both ICIs and non-ICI-immunotherapies in order to establish evidence-based guidelines as support for imaging assessment and clinical decision-making.

## Author contributions

All authors contributed to manuscript drafting, revision and approved the submitted version.

## Supplementary material

Supplementary Figure 1 “Starry sky” appearance. Small echogenic foci representing portal triads and portal venous walls (stars; arrows) can be seen throughout a diffusely hypoechogenic liver parenchyma (sky). This pattern, although non-specific, can be seen with hepatitis.

## Glossary

**Table:**

AKI	acute kidney injury
CAR	chimeric antigen receptor
CMRI	cardiac magnetic resonance imaging
CT	computer tomography
CTCAE	Common Terminology Criteria for Adverse Events
CTLA-4	cytotoxic T-lymphocyte-associated protein-4
FLAIR	fluid-attenuated inversion recovery
GZP	granzyme B-targeted PET tracer
ICI	immune checkpoint inhibitor
irAE	immunotherapy-related adverse events
irRC	immune-related response criteria
irRECIST	immune-related RECIST
LGE	late gadolinium enhancement
mAb	monoclonal antibody
MRCP	magnetic resonance cholangiopancreatography
MRI	magnet resonance imaging
PANCS	primary angiitis of the central nervous system
PD-1	programmed cell death protein-1
PD-L1	PD-1 ligands
PET	positron emission tomography
RECIST 1.1	Response Evaluation Criteria in Solid Tumors version 1.1
STIR	short-tau inversion recovery
SUV	standard uptake value
TTE	transthoracic echocardiography
US	ultrasound
^18^F-FDG	2-deoxy-2-[18F]fluoro-D-glucose
