# Supplementary figures and images for: Imaging assessment of toxicity related to immune checkpoint inhibitors
Source: Front Immunol. 2023 Feb 23;14:1133207. doi: 10.3389/fimmu.2023.1133207 (PMC9995973; doi:10.3389/fimmu.2023.1133207)

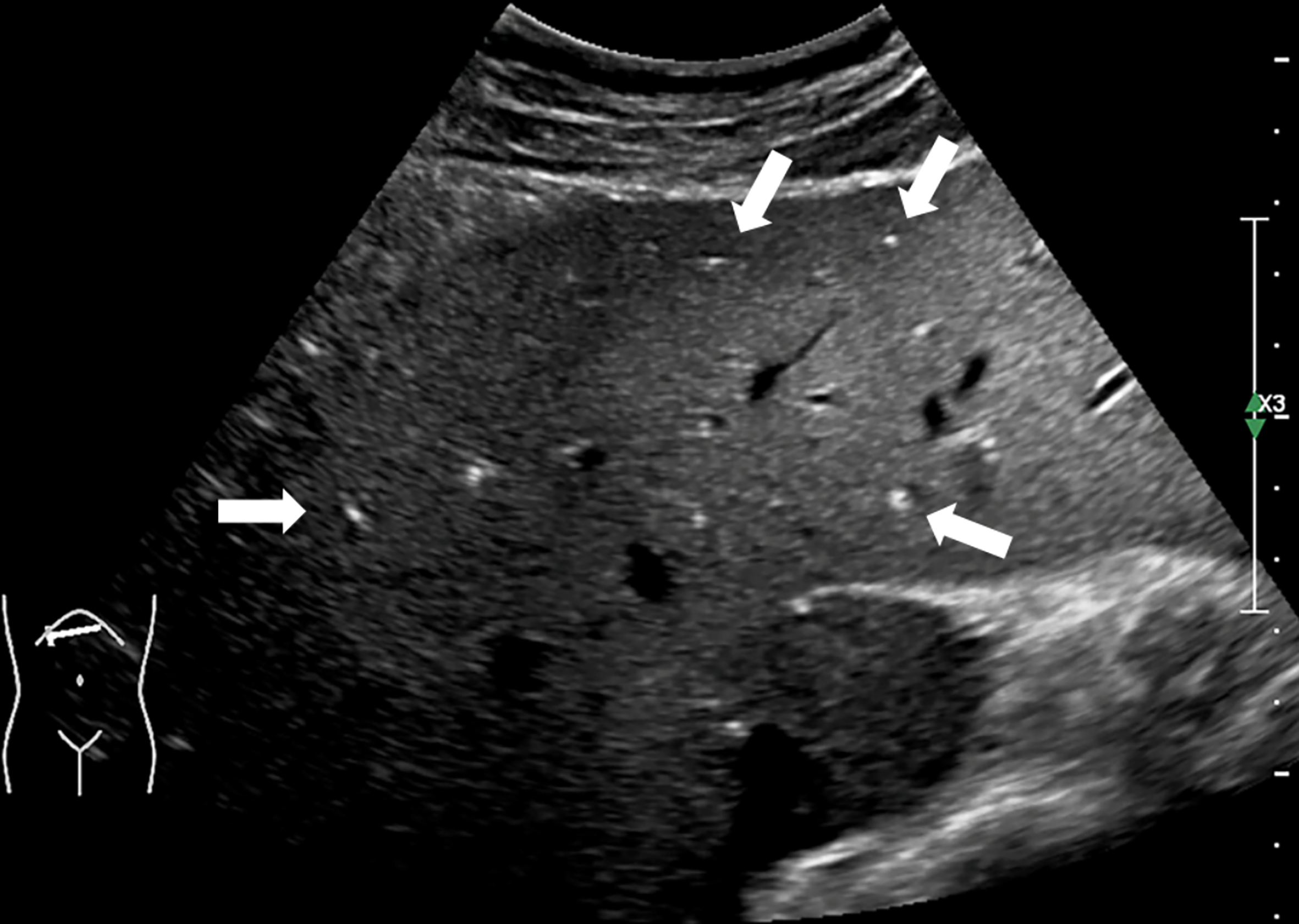

Supplement: Supplementary Figure 1 — “Starry sky” appearance. Small echogenic foci representing portal triads and portal venous walls (stars; arrows) can be seen throughout a diffusely hypoechogenic liver parenchyma (sky). This pattern, although non-specific, can be seen with hepatitis. [file Image_1.tif]
